# Identification and Characterization of Circulating Naïve CD4+ and CD8+ T Cells Recognizing Nickel

**DOI:** 10.3389/fimmu.2019.01331

**Published:** 2019-06-12

**Authors:** Rami Bechara, Sabrina Pollastro, Marie Eliane Azoury, Natacha Szely, Bernard Maillère, Niek de Vries, Marc Pallardy

**Affiliations:** ^1^Inflammation Chimiokines et Immunopathologie, INSERM, Fac. de Pharmacie—Univ. Paris-Sud, Université Paris-Saclay, Châtenay-Malabry, France; ^2^ARC Department of Clinical Immunology and Rheumatology and Department of Experimental Immunology, Academic Medical Center, University of Amsterdam, Amsterdam, Netherlands; ^3^CEA, Institut de Biologie et de Technologies, Université Paris-Saclay, Gif-sur-Yvette, France

**Keywords:** allergy and immunology, T-cell assay, *in vitro* tests, metals hapten, allergic contact dermatitis, naive T cells, repertoire

## Abstract

Allergic contact dermatitis caused by contact sensitizers is a T-cell-mediated inflammatory skin disease. The most prevalent contact allergens is nickel. Whereas, memory T cells from nickel-allergic patients are well-characterized, little is known concerning nickel-specific naïve T-cell repertoire. The purpose of this study was to identify and quantify naïve CD4+ and CD8+ T cells recognizing nickel in the general population. Using a T-cell priming *in vitro* assay based on autologous co-cultures between naïve T cells and dendritic cells loaded with nickel, we were able to detect a naïve CD4+ and CD8+ T-cell repertoire for nickel in 10/11 and 7/8 of the tested donors. We calculated a mean frequency of 0.49 nickel-specific naïve CD4+ T cells and 0.37 nickel-specific naïve CD8+ T cells per million of circulating naïve T cells. The activation of these specific T cells requires MHC molecules and alongside IFN-γ production, some nickel-specific T-cells were able to produce granzyme-B. Interestingly, nickel-specific naïve CD4+ and CD8+ T cells showed a low rate of cross-reactivity with cobalt, another metallic hapten, frequently mixed with nickel in many alloys. Moreover, naïve CD4+ T cells showed a polyclonal TCRβ composition and the presence of highly expanded clones with an enrichment and/or preferentially expansion of some TRBV genes that was donor and T-cell specific. Our results contribute to a better understanding of the mechanism of immunization to nickel and propose the T-cell priming assay as a useful tool to identify antigen-specific naïve T cells.

## Introduction

ACD is a common skin disease affecting up to 20% of the general population (1). It is caused by low-molecular weight chemicals that come into contact with the skin and induce a response by hapten-specific T cells. During the sensitization phase, both CD4+ and CD8+ T cells are activated, in the draining lymph nodes, by skin dendritic cells (DCs) presenting the antigen (2). Two signals are needed to trigger an adaptive immune response: antigen recognition by specific T cell receptors, and a danger signal sensed by DCs leading to their maturation and subsequent activation of T cells (3). A particular feature of contact allergens is their ability to deliver both signals, meaning that they can be considered as “complete allergens” (4, 5). For instance, nickel, which represents the most common contact allergen can directly activate human Toll-like receptor 4 in DCs (6) leading to their maturation (7–11). In contrast to classical haptens that covalently bind to proteins, metallic haptens interact with protein through coordinative bonds generating a specific geometrical configuration (12, 13). Re-exposure to the same hapten initiates the elicitation phase characterized by the rapid recruitment and activation of hapten-specific T cells which leads to the development of clinical symptoms (2, 3).

Nickel-specific memory T cells have been detected in the blood and skin of nickel allergic patients and were shown to proliferate *in vitro* in response to nickel (14–18). Studies performed on nickel-specific memory T cells suggested that nickel recognition by the TCR may occur via both a peptide-dependent (19) and a peptide-independent (20) mechanism. Both mechanisms imply a nickel-mediated bridge between the TCR and MHC molecules with coordinative bonds (12, 21). Despite the high prevalence of concomitant nickel and cobalt allergy (22), nickel-specific memory T cells do not cross-react with cobalt (14–18). Moreover, TCR repertoire analysis of nickel-specific T cells isolated from allergic patients showed in some cases preferential usage of specific TRBV genes (17, 18, 23–25). However, little is known on the factors that determine the strength of the T-cell response such as the naïve T-cell repertoire (26), that can shape the allergic response along with the chemical reactivity of the antigen, the magnitude of the innate inflammatory response and individual-specific factors (27).

The naïve T-cell repertoire is mainly formed by positive and negative selections occurring in the thymus (28). The positive selection allows the survival of T cells that react with MHC molecules, while negative selection eliminates T cells that are strongly activated by self-peptides (28). The specific recognition of an antigen by the T-cell receptor (TCR) of a naïve T cell will lead to its activation and consequently generation of a memory T-cell pool readily available to induce an accelerated recall-response during a second encounter with the antigen (26).

The aim of this study was to identify and characterize the nickel-specific naïve CD4+ and CD8+ T cell repertoire in the general population using an *in vitro* T-cell assay based on the amplification of preexisting antigen-specific T-cells and the evaluation of their antigen specificity. In the present study, we demonstrated the presence of a naïve CD4+ and CD8+ T cell repertoire that is able to recognize nickel in the context of major histocompatibility complex (MHC) molecules and some of these nickel-specific T-cells were able to induce granzyme-B production along with IFN-γ. We also showed that a low frequency of nickel-recognizing naïve CD4+ T cells can cross-react with cobalt. Moreover, we used high-throughput next generation sequencing to explore the TCR repertoire of the nickel-specific naïve T-cells. Nickel-recognizing naïve CD4+ T cells showed a polyclonal TCRVβ composition and the presence of highly expanded clones with an enrichment and/or preferentially expansion of some TRBV genes that was donor and T-cell specific. Our results contribute to a better understanding of the mechanism of immunization to nickel by identifying nickel-specific T cells in the naïve repertoire which share the same characteristics previously described for their memory analogs isolated from allergic patients.

## Materials and Methods

### Blood Collection

Blood was collected from the Etablissement Français du Sang (EFS, Rennes, France) from anonymous healthy donors (these will be named “donors” in the manuscript) after informed consent and following EFS guidelines. Around 300–350 mL of blood from 20 different donors were used in this study. Peripheral blood mononuclear cells (PBMCs) were isolated by density centrifugation on a Ficoll gradient (lymphocyte separation medium LSM1077; PAA, Les Mureaux, France).

### Generation of Naïve CD4+ and CD8+ T-Cells Recognizing Nickel

Monocyte-derived dendritic cells (DCs) were generated from monocytes isolated from PBMC by magnetic-positive selection with MiniMacs separation columns and anti-CD14 antibodies coated on magnetic beads (Miltenyi Biotec, Bergisch Gladbach, Germany) after 5 days of culture in the presence of GM-CSF (550 U/ml) and IL-4 (550 U/ml) (Miltenyi Biotec, Bergisch Gladbach, Germany) in RPMI 1640 (Gibco Invitrogen, Villebon sur Yvette, France) supplemented with 10% heat inactivated fetal bovine serum (FBS, Biowest, France), 1% sodium pyruvate and 1% penicillin/streptomycin (Gibco Invitrogen) at 37°C in humidified air containing 5% CO_2_. Naïve CD4+ T cells were isolated from PBMCs by negative selection using specific microbeads (Miltenyi Biotec). Naïve CD8^+^ T cells were isolated from PBMCs based on a two-step procedure using specific microbeads (Miltenyi Biotec). Percentage of CD4+CD45RA+CCR7+ and CD8+CD45RA+CCR7+ T-cells (>92%) was evaluated using flow cytometry (FACSCalibur, BD Biosciences). Antibodies for surface staining against CD4 (clone RPA-T4; BD Biosciences), CD8 (clone RPA-T8; BD Biosciences), CD45RA (clone REA562; Miltenyi Biotech) and CCR7 (clone REA546; Miltenyi Biotech) were used. For T-cell stimulation, DCs were loaded with nickel sulfate (NiSO4) (500 μM; Sigma-Aldrich, St. Louis, USA) for 24 h. DCs were then washed and 10^4^ cells were added to 10^5^ autologous naïve CD4+ or CD8+ T cells per round-bottom micro well-containing 200 μl Iscove's modified Dulbecco's medium (IMDM) supplemented with 10% human AB serum (Lonza, Basel, Switzerland), 11,000 U/ml of rh-IL-6 and 10 ng/ml rh-IL-12 (R&D Systems). T cells were restimulated on days 7, 14, and 21, with autologous DCs loaded with NiSO4, rh-IL-2 (10 U/ml) and rh-IL-7 (5 ng/ml) (R&D Systems). In the case of naïve CD8+ T cells, rhIL-15 (25 ng/ml) was also added at day 21. Specificity of the T-cell lines was investigated using IFN-γ ELISPOT assay at day 27. T-cell stimulation with KLH (Keyhole Limpet Hemocyanin; Thermo Fisher Scientific) was used as a positive control. The viability of randomly chosen T cells, after 3 round of stimulation, was assessed by PI (propidium iodide) staining (Supplementary Data 1).

### ELISpot/Fluorospot Assay

IFN-γ ELISpot and IFN-γ/Granzyme B Fluorospot assays (MabTech) assay was performed, according to the manufacturer's instructions, after 18 h of co-culturing T cells and DCs loaded with NiSO_4_ or KLH. In some experiments, DCs were loaded with CoCl_2_ (500 μM) to address cross-reactivity of nickel-specific T cells. The number of spots was determined using the AID ELISpot Reader system (AID). T-cell lines were considered specific when the spot count was at least two-fold higher in the presence of NiSO_4_-loaded DCs compared to non-loaded DCs. A minimum of 30 spots/well was required for the analysis to be considered valid.

### Requirement for MHC Class I and II Molecules

Anti-human MHC class II antibodies (Abs): anti-human HLA-DP, DQ and DRα blocking antibodies (10 μg/ml; a kind gift from Dr. Bernard Maillère) and anti-human MHC class I Abs (10 μg/ml; Clone DX17; BD France) were used to determine the role of the MHC molecules in T-cell responses to nickel. DCs were pre-treated for 1 h with the different MHC class II or class I Abs and then stimulated with NiSO_4_ (500 μM) for 24 h before starting the autologous co-culture assay. Implication of MHC molecules in the T cell response to nickel was assigned to wells in which the number of IFN-γ spots was 2-fold lower in the presence of MHC blocking antibody than in its absence.

### High-Throughput Sequencing

CD4+ naïve T-cell lines were selected for high-throughput sequencing of the T cell receptor β chain (TCRβ) repertoire. Cell pellets were snap frozen in liquid nitrogen and store at −80°C until RNA extraction. Due to the small cells numbers, 10^5^ (TCR-negative) HEK293T cells were added to each samples before starting the RNA isolation procedure, performed following manufacturer's instructions (QIAGEN, Hilden, Germany). Complementary DNA (cDNA) synthesis and amplification of the TCRβ repertoire were performed as previously described (29, 30). Briefly, a linear amplification was performed using a mix of primers covering all the functional TCRβ variable genes. After purification, the amplification product was used in a normal PCR to obtain amplicons spacing from the TCRβ variable region to the TCRβ constant region. Amplicons were purified, quantified, and prepared for sequencing according to the sequencing platform manufacturer's manual (Illumina MiSeq, San Diego, California, USA).

### CDR3 Sequence Analysis of Naïve CD4+ T Cells

A total of 305,683 reads were retrieved from the sequencing platform. After quality check and filtering, a total of 15,000 randomly selected reads per sample were used for repertoire analysis. The bioinformatic pipeline used to extract TCRβ sequences was described previously (29, 30). In short, reads are “fingerprinted” based on the V-J-CDR3 identified in the sequence (V, Variable gene; J, Joining gene; CDR3, Complementary Determining Region 3). TCRβ sequences with the same unique fingerprint were regarded as a *clone*. The frequency of each clone was calculated based on the total amount of reads. Clones with frequencies above 0.5% of the total repertoire were considered to be Highly Expanded Clones (HECs).

### Statistical Analysis

The frequency of naïve T cells-recognizing nickel was estimated using the Poisson distribution according to the following formula:

$$\text{Frequency}=\frac{-\text{Ln }(\text{Number of negative T}-\text{cell lines }\backslash \text{ Total number of T ​}-\text{ ​cell lines tested})}{\text{Number of T​ }-\text{ ​cells per well}}$$

## Results

### Detection of Nickel-Specific Naïve CD4+ and CD8+ T-Cell Lines

**Figure 1:**
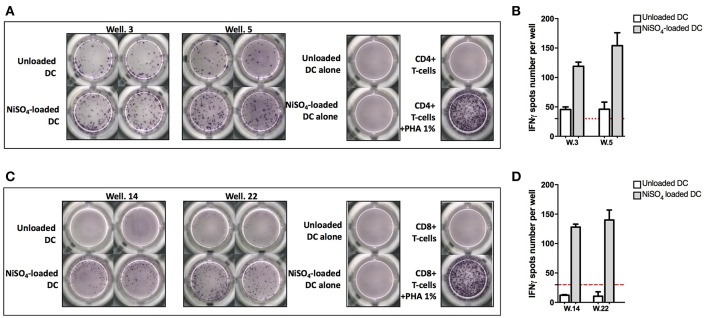
Naïve CD4+ and CD8+ T-cell lines can recognize nickel. **(A)** IFN-γ ELISpot response of naïve CD4+ T cells from donor PR4 stimulated with unloaded DC or DC loaded with NiSO_4._ **(B)** Spots count for naïve CD4+ T cells specific to nickel from donor PR4. Dashed line represents the minimum required spots count (count = 30) for the analysis to be considered acceptable. **(C)** IFN-γ ELISpot response of naïve CD8 + T cells from donor PR13 stimulated with unloaded DC or DC loaded with NiSO_4_. **(D)** Spots count for naïve CD8 + T cells specific to nickel from donor PR13. Dashed line represents the minimum required spots count (count = 30) for the analysis to be considered acceptable. PHA: Phytohemagglutinin.

To identify naïve T cells that are able to recognize nickel, naïve CD4+ and CD8+ T cells, expressing CD45RA and CCR7, were isolated from PBMCs and co-cultured with autologous DCs loaded with NiSO_4_ (500 μM). Weekly re-stimulation of the T cells was performed by addition of fresh autologous DCs loaded with NiSO_4_ for a total of 3 re-stimulation rounds. At day 27, each independent T-cell line (T cells present in a single well) was evaluated for its ability to recognize nickel using an IFN-γ ELISpot assay where each T-cell line was stimulated with unloaded DCs or NiSO_4_-loaded DCs. Results from donors PR4 for naïve CD4+ T cells and PR13 for naïve CD8+ T cells are shown as a representative result of the 11 and 8 donors tested, respectively (Figure 1). For donor PR4, two naïve CD4+ T-cell lines (well 3 and 5; PR4.03 and PR4.05) were found specific to nickel among a total of 90 CD4+ naïve T-cell lines tested (Figures 1A,B). For donor PR13, two naïve CD8+ T-cell lines (well 14 and 22; PR13.14 and PR13.22) were found specific to nickel among a total of 30 naïve CD8+ T-cell lines tested (Figures 1C,D).

### Quantification of the Frequency of Blood-Circulating Nickel-Recognizing naïve CD4+ and CD8+ T Cells

**Figure 2:**
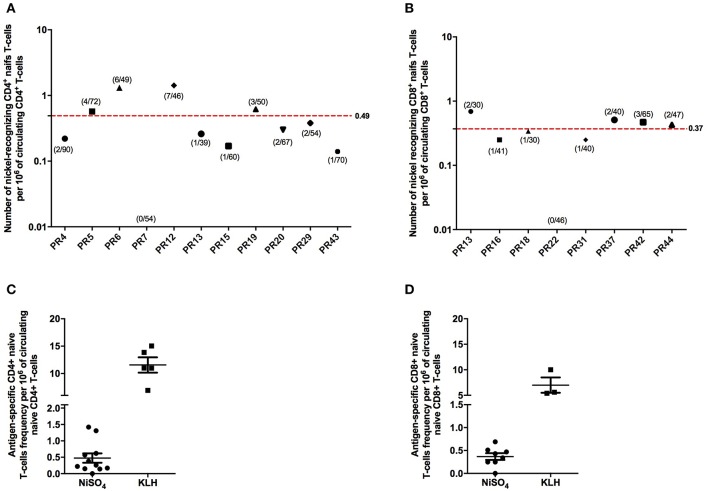
Frequency of human naive CD4 + and CD8 + T cells recognizing nickel. Frequency of human naïve CD4+ **(A)** and CD8+ **(B)** T cells specific to nickel. Frequency was calculated for each donor using the Poisson distribution law. On the x-axis is represented the donor's number. For the different donors tested, the number of T-cell lines found specific for nickel out of the number of T-cell lines tested is indicated in brackets. **(C,D)** Comparison of the nickel-specific T-cell frequency to the KLH-specific T-cell frequency measured on different donors.

Naïve CD4+ and CD8+ T cells that were able to recognize nickel were detected in, respectively, 10/11 and 7/8 healthy donors tested (Figure 2). PR7 for naïve CD4+ T cells and PR22 for naïve CD8+ T cells were the only non-responder donors. We then used these results to calculate the frequency of preexisting nickel-recognizing naïve CD4+ and CD8+ T cells for each donor using the Poisson distribution law. For nickel-specific naïve CD4+ T cells, the 10 responding donors exhibited a frequency varying from 0.14 to 1.42 cells per million of circulating naïve CD4+ T cells (Figure 2A). The mean frequency for the 11 tested donors was 0.49 nickel-recognizing naïve CD4+ T cells per million of circulating naïve CD4+ T. The frequency for KLH-specific naïve CD4+ T cells used as a positive control was 11.57 (Figure 2C). For naïve CD8+ T cells, the mean frequency for the 8 tested donors was 0.37 nickel-recognizing naïve CD8+ T cells per million of circulating naïve CD8+ T cells (Figure 2B). The frequency for KLH-specific naïve CD8+ T cells used as a positive control was 7 (Figure 2D). Spots count for nickel-recognizing naïve CD4+ and CD8+ T cells from each single tested donor is shown in Supplementary Datas 2, 3.

### Nickel-Specific T-Cells Produce Granzyme B

**Figure 3:**
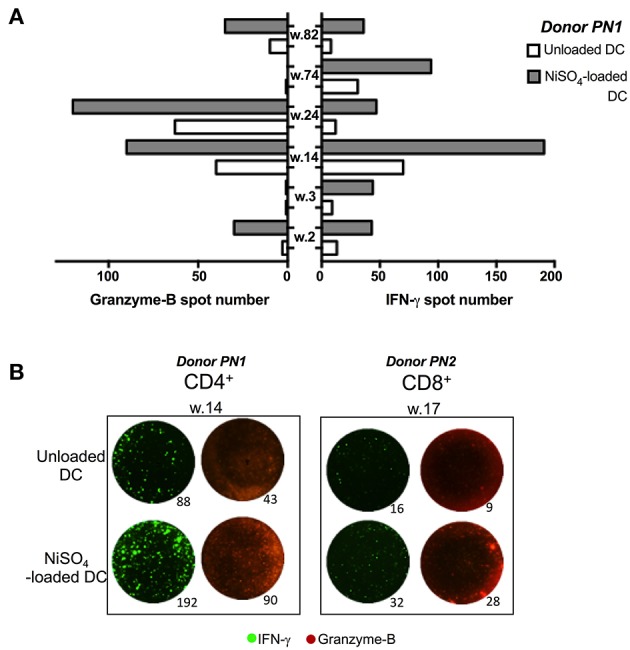
Granzyme B production from CD4 + and CD8 + T cells recognizing nickel. **(A)** Granzyme-B (Grz-B)/ IFN-γ spots count for nickel-specific CD4+ T-cells, from donor PN1, stimulated with unloaded DC or DC loaded with NiSO_4_. **(B)** Representative figure from donor PN1 (CD4+; well number 14) and PN2 (CD8+; well number 17) performed using an IFN-γ/Grz-B fluorospot assay.

To further characterize nickel-specific CD4+ and CD8+ T-cells response, additional experiments were performed on nickel-specific T cells, identified in a first ELISpot assay, using IFN-γ and granzyme B (Grz-B) secretion as readouts. Alongside IFN-γ production, granzyme B was released from 4 nickel-specific CD4+ T-cells from donor PN1 out of 6 nickel-specific CD4+ T-cells identified in the first ELISpot assay (Figures 3A,B). Moreover, one CD8+ T-cell (well 17) was found specific to nickel among a total of 30 CD8+ naïve T-cell lines tested from donor PN2. This CD8+ T-cell line was also able to produce Grz-B at least two time more than the unloaded DCs (Figure 3B).

### MHC Class II and I Molecules Are Required for T-Cell Activation

**Figure 4:**
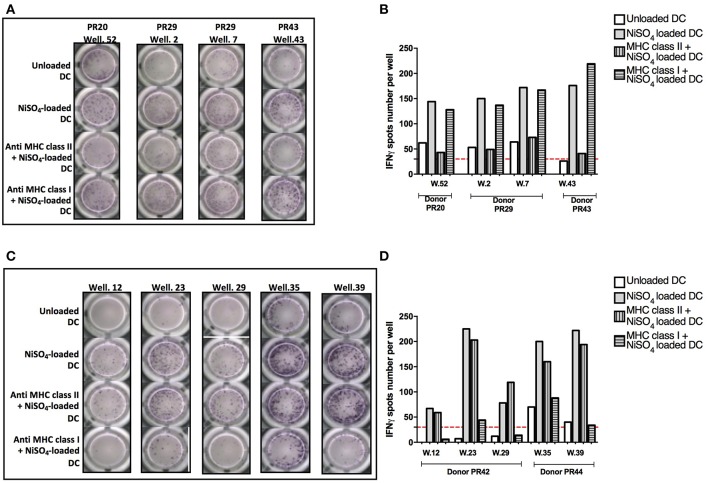
Implication of MHC class II and I molecules in nickel-specific T-cell response. **(A)** IFN-γ ELISpot response for naïve CD4+ T cells from donors PR20, PR29, and PR43 stimulated with unloaded DC or DC loaded with NiSO_4_ or DC loaded with MHC blocking antibodies prior to nickel treatment. **(B)** Spots count for naïve CD4+ T cells specific to nickel from donors PR20, PR29, and PR43 stimulated with unloaded DC or DC loaded with NiSO_4_ or DC loaded with MHC blocking antibodies prior to nickel treatment. Dashed line represents the minimum required spots count (count = 30) for the analysis to be considered acceptable. **(C)** IFN-γ ELISpot response of naïve CD8+ T cells from donors PR42 and PR44 stimulated with unloaded DC or DC loaded with NiSO_4_ or DC loaded with MHC blocking antibodies prior to nickel treatment. **(D)** Spots count for naïve CD8+ T cells specific for nickel from donors PR42 and PR44 stimulated with unloaded DC or DC loaded with NiSO_4_ or DC loaded with MHC blocking antibodies prior to nickel treatment. Dashed line represents the minimum required spots count (count = 30) for the analysis to be considered acceptable.

To determine whether the T cell response to nickel is mediated by MHC molecules, we tested the capacity of blocking antibodies specific for MHC class I (A, B, and C) and II (DP, DQ and DR) molecules to inhibit the activation of nickel-specific T-cell lines. Purified naïve CD4+ and CD8+ T cells were seeded in multiple wells and stimulated weekly by autologous DCs previously loaded with nickel to enrich the co-cultures in antigen-specific T cells. After 3 rounds of stimulation, each independent T-cell line was evaluated for its specificity using IFN-γ ELISpot. Four naïve CD4+ T-cell lines specific to nickel were identified from three different donors (PR20, PR29, and PR43) in the first ELISpot assay. In a second ELISpot assay, we confirmed the positivity of these T-cell lines for nickel and we showed that the response was dependent on MHC class II molecules but not on MHC class I molecules (Figures 4A,B). Likewise, we tested five naïve CD8+ T-cell lines specific to nickel derived from two different donors (PR42 and PR44) in a second ELISpot assay. Our results showed that the activation of these naïve CD8+ T cells was dependent on MHC class I molecules but not on MHC class II molecules (Figures 4C,D).

### Cross-Reactivity of Nickel-Recognizing Naïve T-Cells With Cobalt

**Figure 5:**
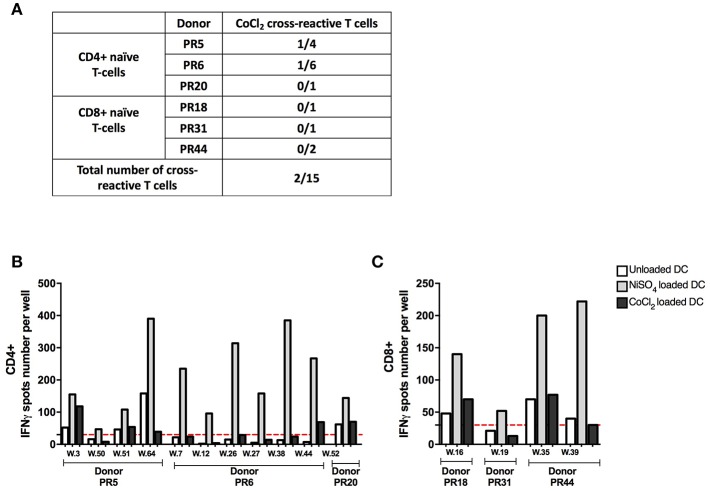
Cobalt cross-reactivity of nickel-recognizing naïve CD4+ and CD8+ T cells. **(A)** Number of cross-reactive T-cell lines among nickel-specific T cells from different donors. **(B)** Spots count for naïve CD4+ T cells specific to nickel from donors PR5, PR6, and PR20 stimulated with unloaded DC or DC loaded with NiSO_4_ or DC loaded with CoCl_2_. **(C)** Spots count for naïve CD8+ T cells specific to nickel from donors PR18, PR31, and PR44 stimulated with unloaded DC or DC loaded with NiSO_4_ or DC loaded with CoCl_2._ Dashed line represents the minimum required spots count (count = 30) for the analysis be considered acceptable. w, well-number.

Nickel and cobalt are constituents of many alloys and a high prevalence of concomitant nickel and cobalt allergy can be observed in the general population (22). We therefore tested the cross-reactivity of the identified nickel-recognizing naïve CD4+ and CD8+ T-cells lines to cobalt. (Supplementary Data 4). Purified naïve CD4+ and CD8+ T cells were seeded in multiple wells and stimulated weekly by autologous DCs previously loaded with nickel to enrich the co-cultures in antigen-specific T cells. After three rounds of stimulation, each independent T-cell line was evaluated for its specificity using IFN-γ ELISpot. A total of 15 nickel-recognizing naïve T-cell lines identified by a first ELISpot assay from six different donors were tested in a second ELISpot assay for their cross-reactivity to cobalt (Figure 5A). Our results showed that only two nickel-recognizing naïve CD4+ T-cell lines (PR5.03 and PR6.44) but no naïve CD8+ T-cells were also able to recognize cobalt (Figures 5B,C).

### TCRβ Repertoire Analysis of Nickel-Recognizing Naïve CD4+ T Cells

**Figure 6:**
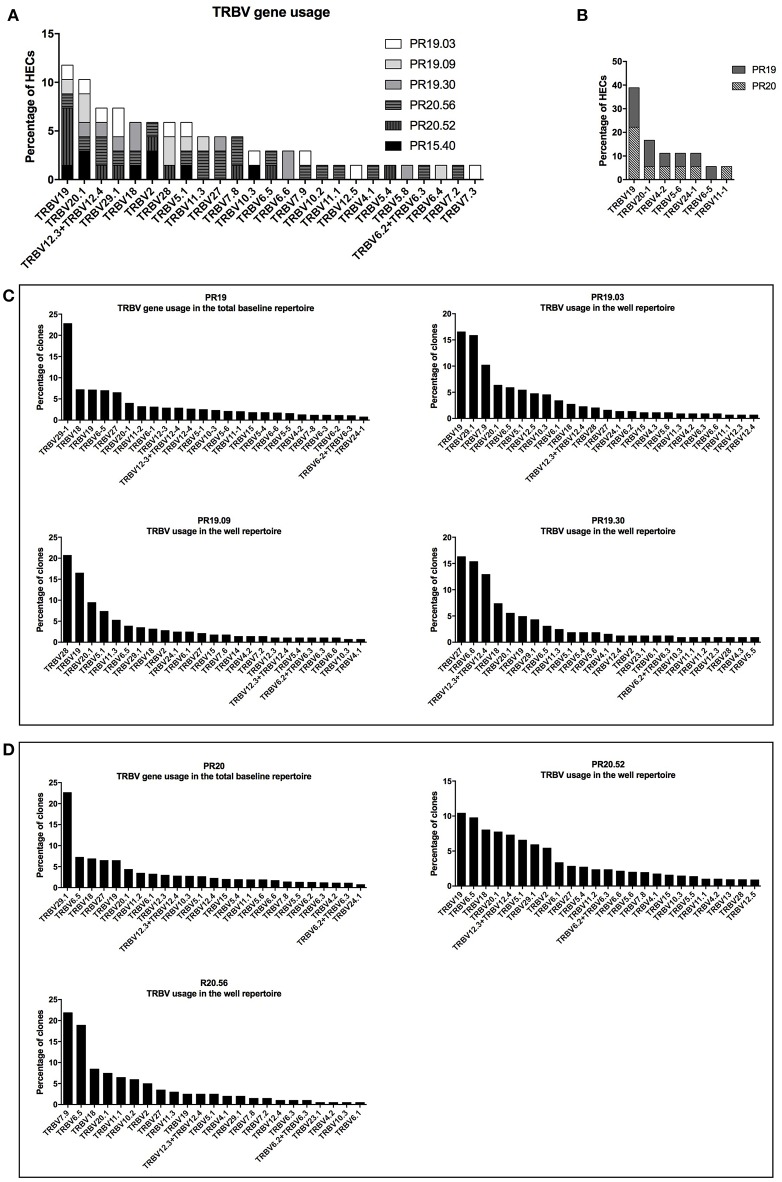
TCRβ repertoire analysis of nickel-recognizing naïve CD4+ T-cell lines. TCRβ variable gene usage of the highly expanded clones (HECs) detected in the six nickel-specific T-cell lines analyzed **(A)** and in the sorted naïve CD4+ T-cells from donors PR19 and PR20 **(B)**. TCRβ variable gene usage in the total repertoire pre- and post-stimulation in donors PR19 **(C)** and PR20 **(D)**. The percentage of clones (or HECs) carrying a particular TCRβ variable gene (TRBV) is depicted on the y-axis. All the genes names follow the IMGT nomenclature.

Previous studies indicated that nickel-specific memory T cells derived from nickel-allergic patients preferentially express distinct TCR Vβ chains (18, 24, 25). To gain more insight into the clonal expansion of nickel-specific naïve T cells, six nickel-recognizing naïve CD4+ T-cell lines from three different donors were randomly selected for TCRβ repertoire analysis using high-throughput next generation sequencing. All T-cells lines analyzed showed a polyclonal TCRβ repertoire composition and an elevated clonal expansion, with 93% (median, IQR: 92–96%) of the TCRβ repertoire being occupied by highly expanded clones (HECs, clonal frequency >0.5% of the total repertoire) (Supplementary Datas 5a,b). The most represented TCRβ variable gene among the highly expanded clones of all T-cell lines analyzed was TRBV19, followed by TRBV20.1 (11.8 and 10.3% respectively of the total HECs) (Figure 6A). To verify whether the skewed usage of Vβ genes observed in the TCR repertoire of nickel-specific T-cell lines is a nickel-induced or a donor-related effect, we analyzed the TRBV gene usage of sorted unstimulated naïve CD4+ T cells from two donors (PR19 and PR20). Prior to perform the Vβ usage analysis, our results showed that the baseline repertoires had a higher diversity index compared to the correspondent post-culture wells-repertoires (Supplementary Data 6). In addition, the post-stimulation sample's diversity decreases even more when calculating the Shannon and/or Simpson index compared to Richness (i.e., when also clonal frequency is considered to calculated diversity). These results indicate that the baseline repertoires is composed of more different TCR clones while fewer and more expanded TCR clones dominate the post-stimulation repertoire, probably due to enrichment for nickel-specific TRBV clones. Moving to the V gene usage, the most used TRBV gene among all the detected HECs of the unstimulated naïve CD4+ sorted populations was TRBV19 (Figure 6B), indicating that this Vβ gene is already highly represented prior to nickel stimulation in two tested donors. However, if we consider the entire clonal repertoire (and not only the highly expanded fraction), changes in TRBV usage are observed after nickel-priming (Figures 6C,D). Indeed, particular TRBV genes become highly represented in the repertoire of nickel-specific naïve T cell lines in a donor and T-cell dependent manner: TRBV19 and TRBV7.9 for PR19.03; TRBV28 and TRBV19 for PR19.09; TRBV27 and TRBV6.6 for PR19.30 (Figure 6C), TRBV20.1 for PR20.52 and TRBV7.9 and TRBV6.5 for PR20.56 (Figure 6D). Moreover, highly expanded clones (HECs) carrying these particular TRBV genes were found in all T cell lines tested (Supplementary Datas 5c,d). Inversely, the usage of some TRBV genes such as TRBV29.1 decreased from 22.5% in the pre-stimulation repertoire, to <5% in the post-stimulation for some of the analyzed nickel-specific T-cells lines (PR19.09, PR19.30, and PR20.56) (Figures 6C,D).

## Discussion

ACD is a common T cell-mediated inflammatory skin disease and is regarded as a prototype of delayed hypersensitivity (type IV hypersensitivity), as classified by Gell and Coombs (3). Nickel-specific T cells have been previously detected in the blood and skin of nickel allergic patients (14–18) and the response of nickel-specific memory T cells has been well-characterized. Different groups have isolated nickel-specific T-cell clones from allergic patients *in vitro* (18, 31, 32). These clones showed heterogeneous responses depending on whether their TCR was recognizing nickel bound to the peptide-MHC molecule complex (clone ANi-2.3) (19) or whether no specific MHC-bound peptide was required (clone SE9) (20). However, much less is known concerning the naïve T-cell repertoire that can recognize nickel. The objectives of this work were to identify naïve CD4+ and CD8+ T cells recognizing nickel in the general population, to characterize their TCRβ repertoire and to study the cross-reactivity to cobalt, a common metal allergen usually found with nickel in many alloys (22).

Our results showed the presence of a naïve CD4+ T-cell repertoire to nickel. This is in concordance with previous reports showing that nickel can activate CD4+ T cells even from non-allergic individuals (33–36). The mean frequency of nickel-recognizing naïve CD4+ T cells was 0.49 per million of circulating naïve T cells. The result for CD4+ T cells was very similar to the one analyzed by linear regression where a frequency of <1 nickel-specific cell per 2 ×10^6^ T cells was found (33). Of particular interest in our study is the finding that a naïve CD8+ T-cell repertoire for nickel could also be detected in almost all the tested donors with a mean frequency of 0.37. Previous studies using the lymphocyte proliferation assay as the end-point of a co-culture assay between antigen presenting cells (APC) and T cells in the presence of nickel, could not detect CD8+ T-cells proliferation in non-allergic donors (34, 35). This discrepancy could be due to a better sensitivity of our T-cell priming assay. In fact, the repetitive restimulations of naïve T cells by nickel-loaded autologous DCs allows progressive expansion of nickel-specific T cells already present at low frequency and therefore facilitates their detection. Multiple similar strategies based on DC/T cell co-cultures and restimulations have already been used to characterize the naïve T-cell response to a variety of drugs, chemicals and therapeutic antibodies (37–43). Moreover, CD8+ T-cells activation might also be prevented by regulatory mechanisms. In our model, immunomodulatory CD25+ and CD56+ cells were removed during the selection process of naïve T-cells. Our data are in line with other studies showing that the depletion of CD25+ and CD56+ cells in peripheral blood lymphocytes improves the *in vitro* detection of hapten-specific T cells (41, 44).

In this study, the frequency of nickel-recognizing naïve T cells was very low as expected for hapten-specific T cells in non-sensitized individuals (33, 39). Previous works performed on nickel allergic patients showed a frequency of nearly 100 to 1,000-fold higher than our findings (34). However, the frequencies of naïve T cells recognizing benzylpenicillin (BP) (39, 45, 46) and immunogenic therapeutic antibodies (40) in non-immunized individuals were in the same range as those found in our study. It is important to note that the frequency of nickel-recognizing naïve T cells that we identified could still be an underestimation because of the use of nickel-loaded DCs, whereas it was shown that the activation of some nickel-specific T-cell lines (such as clone SE9) requires the persistent presence of nickel in the culture medium (20). Moreover, T memory stem (Tscm) cells are a rare subset of memory lymphocytes, distinguished from naïve T-cells based on CD95 expression, with the ability to rapidly proliferate and release inflammatory cytokines in response to antigen re-exposure while naïve T cells remained relatively quiescent (47). In our conditions, at least three restimulations with antigen-loaded DCs are needed to detect CD8+ or CD4+ positive cells based on IFN-γ production. Thus, we believe that our culture conditions are not in favor of the detection of Tscm.

To explore the molecular requirement for nickel to stimulate specific T cells, we performed MHC blocking experiments. Our observation that the blocking of the MHC class II molecules alters the CD4+ response but not the CD8+ response, whereas the blocking of the MHC class I molecules alters CD8+ response is in agreement with the specific activation of each T-cell subtype and the requirement for MHC molecules.

It is well-known that ACD to different metals can occur simultaneously, and that a high prevalence of concomitant nickel and cobalt allergy can be found in the general population (22). In our study, most nickel-specific naïve T cells tested showed a high specificity to nickel since they were not able to recognize cobalt, another metallic hapten belonging to the same period as nickel. In fact, nickel and cobalt have different coordination geometries in their protein bound state (13) that may explain why cobalt could not be recognized by nickel-specific TCR. Interestingly, also nickel-specific memory T-cell clones derived *in vitro* from allergic patients did not proliferate in response to cobalt (18, 48). Taken together, these results indicate that nickel-specific naïve T cells isolated from non-sensitized individuals showed the same behavior as their memory analogs isolated from allergic patients.

To further characterize nickel-specific T cells, we assessed the clonal expansion of nickel-specific naïve T cells by exploring their TCR Vβ repertoire before and after nickel priming using high-throughput next generation sequencing. In comparison with flow cytometric or spectratyping approaches commonly used to characterize the TCR Vβ repertoire of memory nickel-specific T cells (18, 25), high-throughput sequencing allows greater sequencing depth and significantly more accurate quantification of TCR clonotype abundance. It should be noted that in our study we used the IMGT gene nomenclature (49) which has been approved by the HUGO Nomenclature Committee. For example, the TRBV19, TRBV20.1, TRBV27, TRBV7.9, and TRBV28 in our study were previously termed TCRVβ17, TCRVβ2, TCRVβ14, TCRVβ6S5, and TCRVβ3, according to the nomenclature of Wei et al., respectively (50). Our results showed that nickel-specific naïve CD4+ T cells analyzed after priming/restimulation showed a different TRBV gene usage distribution compared to naïve sorted human T cells prior to stimulation, indicating a skewing of the TCRβ repertoire toward specific TRBV genes in a T-cell and donor dependent manner. These results could indicate that multiple TCRs may be involved in nickel recognition. Interestingly, nickel-specific CD4+ T cells showed an enrichment of some TRBV genes which were also detected in nickel-specific memory T cells isolated from allergic patients. For instance, TRBV27, TRBV28, and TRBV6.5 were also detected in nickel-specific T cells isolated from allergic patients (18, 25). Moreover, it was demonstrated that TRBV19 gene's over-expression was correlated with the individuals reactivity to nickel (17, 18, 24, 51). However, we could not conclude that nickel priming induces enrichment and/or preferentially expansion of TCRβ clones carrying TRBV19 since this gene was highly represented prior to stimulation.

In summary, our results showed the existence of a naïve CD4+ and CD8+ T cells repertoire for nickel in the general population. Using an *in vitro* T-cell assay, we were able to estimate the frequency of nickel-recognizing naïve T cells in the circulation. These nickel-specific naïve CD4+ and CD8+ T cells were shown to recognize nickel in the context of major histocompatibility complex (MHC) class II and I molecules, respectively. Moreover, a low percentage of nickel-recognizing naïve CD4+ T cells could cross-react with cobalt. Nickel-recognizing naïve CD4+ T cells showed a polyclonal TCR Vβ composition with a skewed repertoire toward some TRBV genes found earlier in T-cells from allergic patients. Our results contribute to a better understanding of the mechanism of immunization to nickel by identifying nickel-specific T cells in the naïve repertoire which share the same characteristics previously described for their memory analogs isolated from allergic patients. In comparison to CD69 expression alone which does not seem to be strictly antigen specific and may strongly overestimate the frequencies of antigen-specific T cells (Supplementary Data 7), our results propose the T-cell priming assay as a useful tool to identify antigen-specific naïve T cells. Thus, our experimental approach presented in this study could be used as a prototype to develop an *in vitro* assay for the identification of skin sensitizing chemicals, where low frequencies of antigen-specific T cells occurring in the naïve T cell pool are expanded by antigen-specific priming and restimulation. Moreover, the preexistence of a naïve T-cell repertoire for nickel in almost all the tested donors further supports the hypothesis that most people can be sensitized to nickel but only a few can mount an allergic response due to additional factors such as the rate of exposition or the disturbance of tolerance (52). However, the correlation between the size of the naïve T-cell repertoire and the potential potency of the chemicals as described for the immunogenicity of therapeutic antibodies (40) will need further investigation.

## Ethics Statement

Blood was collected from the Etablissement Français du Sang (EFS, Rennes, France) from anonymous healthy donors (these will be named donors in the manuscript) after informed consent and following EFS guidelines.

## Author Contributions

RB performed experiments on naïve T cells, analyzed the data and wrote the manuscript. MA performed experiments on naïve T cells. SP and NdV performed the TCR V repertoire analysis. BM participated in study design. MP designed and supervised the conduct of the study and participated in data analysis and interpretation. MP, NdV, SP, NS, and BM critically reviewed the manuscript and all authors approved the final version.

### Conflict of Interest Statement

The authors declare that the research was conducted in the absence of any commercial or financial relationships that could be construed as a potential conflict of interest.

## Supplementary Material

Supplementary Data 1 Gating strategy and cell viability for MoDC/T-cells co-culture. **(a)** IP (propidium iodide) staining of MoDC and CD8 + T cells after 7 days of coculture. **(b)** IP staining of CD8 + T cells detected after 3 round of stimulation. Purified naïve CD8+ T were seeded in multiple wells and stimulated weekly by autologous DCs previously loaded with nickel. After three rounds of stimulation, the specificity of the T-cell lines was tested by IFN-γ Elispot assays. One positive T-cell line was randomly chosen and its viability was assessed with IP staining.

Supplementary Data 2 Spots count for nickel-recognizing naïve CD4+ T cells. Spots count for naïve CD4+ T cells specific to nickel from donors PR4, PR5, PR6, PR12, PR13, PR15, PR19, PR20, PR29, and PR43. Purified CD4+ T cells from each donor were seeded in multiple wells and stimulated weekly by autologous DCs previously loaded with nickel. After three rounds of stimulation, the specificity of the T-cell lines (each well-containing the expanded T cells) was tested by IFN-γ ELISpot assays. Dashed line represents the minimum required spots count (count = 30) for the analysis to be considered acceptable. Each graph represent one donor and donor number are present on the top of each graph.

Supplementary Data 3 Spots count for nickel-recognizing naïve CD8+ T cells. Spots count for naïve CD4+ T cells specific to nickel from donors PR13, PR16, PR18, PR31, PR37, PR42, and PR44. Purified CD8+ T cells from each donor were seeded in multiple wells and stimulated weekly by autologous DCs previously loaded with nickel. After three rounds of stimulation, the specificity of the T-cell lines (each well-containing the expanded T cells) was tested by IFN-γ ELISpot assays. Dashed line represents the minimum required spots count (count = 30) for the analysis to be considered acceptable. Each graph represent one donor and donor number are present on the top of each graph.

Supplementary Data 4 IFN-γ ELISPOT response of naïve T-cells. Purified naïve CD4+ or CD8+ T cells from each donor were seeded in multiple wells and stimulated weekly by autologous DCs previously loaded with nickel. After three rounds of stimulation, the specificity of the T-cell lines (each well-containing the expanded T cells) was tested by IFN-γ ELISpot assays **(a)** IFN-γ ELISpot response of naïve CD4+ T cells from donors PR5, PR6, and PR20 stimulated with unloaded DC, DC loaded with NiSO_4_ or DC loaded with CoCl_2_. **(b)** IFN-γ ELISpot response of naïve CD8+ T cells from donors PR18, PR19, and PR44 stimulated with unloaded DC, DC loaded with NiSO_4_ or DC loaded with CoCl_2_.

Supplementary Data 5 TCRβ V gene usage of HECs based on clonal frequency. **(a)** Scatter dots plot representing the clonal distribution of the TCRβ repertoire for the six nickel-specific T-cell lines analyzed. Each dot represents a single clone and its frequency in the total repertoire is depicted on the y-axis as percentage of total reads. The gray dotted line indicates the frequency cut-off of 0.5% for the definition of highly expanded clones (HECs). **(b)** Impact on the total repertoire of clones with clonal frequency below 0.1% (light gray bars), between 0.1 and 0.5% (dark gray bars) and above 0.5% (black bars) in the six nickel-specific T-cell lines analyzed. TCRβ variable gene usage of the HECs in the pre- and post-stimulation repertoire in donor PR19 **(c)** and PR20 **(d)**. The percentage of HECs carrying a particular TCRβ variable gene (TRBV) is depicted on the y-axis. All the genes names follow the IMGT nomenclature.

Supplementary Data 6 Diversity index of the TCRβ repertoire at baseline and in wells after coculture with nickel-loaded DCs. The diversity in the pre- (baseline) and post-stimulation (well) repertoire in donor PR19 and PR20 measured as Richness (alpha = 0), Shannon entropy (alpha = 1) and Simpson index (alpha = 2). On the x-axis, “Well” for donor PR19 includes results from PR19.03, PR19.09, and PR19.30. “Well” for donor PR20 includes results from PR20.52 and PR20.56.

Supplementary Data 7 CD69 expression on naïve CD4+ T cells cocultured with NiSO_4_-loaded DCs or unloaded DCs. Purified naïve CD4+ T cells were seeded in multiple wells and stimulated weekly by autologous DCs previously loaded with nickel to enrich the co-cultures in antigen-specific T cells. After 2 rounds of stimulation, each independent T-cell line was co-cultured with NiSO_4_-loaded DCs or unloaded DCs as a negative control. CD69 expression was evaluated by flow cytometry. 76 naïve CD4+ T-cell lines were evaluated for CD69 expression upon nickel stimulation and the majority of them showed an increase in CD69 expression upon coculture with NiSO_4_-loaded DCs.

## Abbreviations

ACD: Allergic contact dermatitis
CoCl_2_: Cobalt chloride
DC: Dendritic cell
MHC: Major histocompatibility complex
MoDC: Monocyte-derived dendritic cell
NiSO_4_: Nickel sulfate
TCR: T-cell receptor.
