# Supplementary material for: Identification and Characterization of Circulating Naïve CD4+ and CD8+ T Cells Recognizing Nickel
Source: Front Immunol. 2019 Jun 12;10:1331. doi: 10.3389/fimmu.2019.01331 (PMC6582854; doi:10.3389/fimmu.2019.01331)

Supplementary data 1

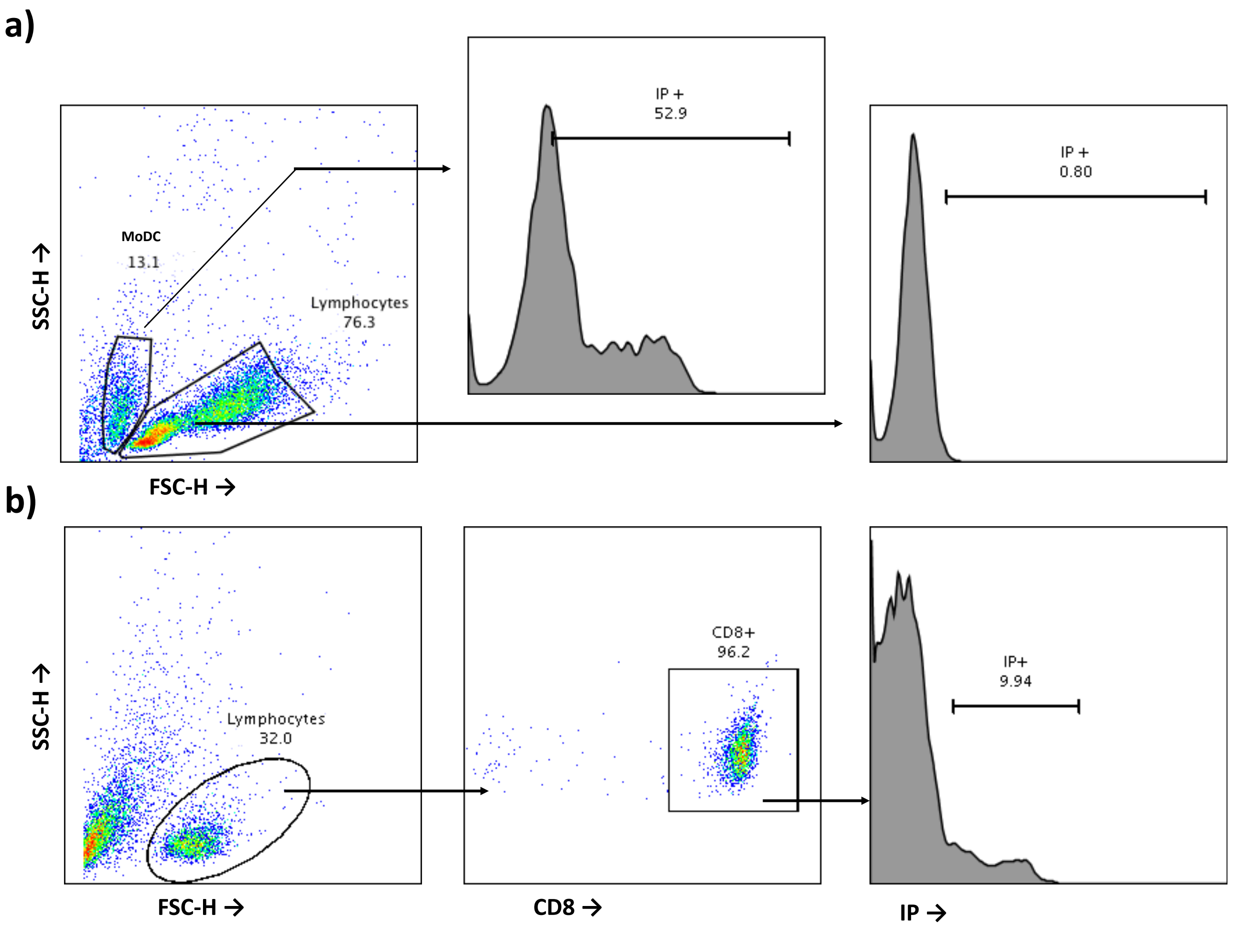

Supplementary data 2

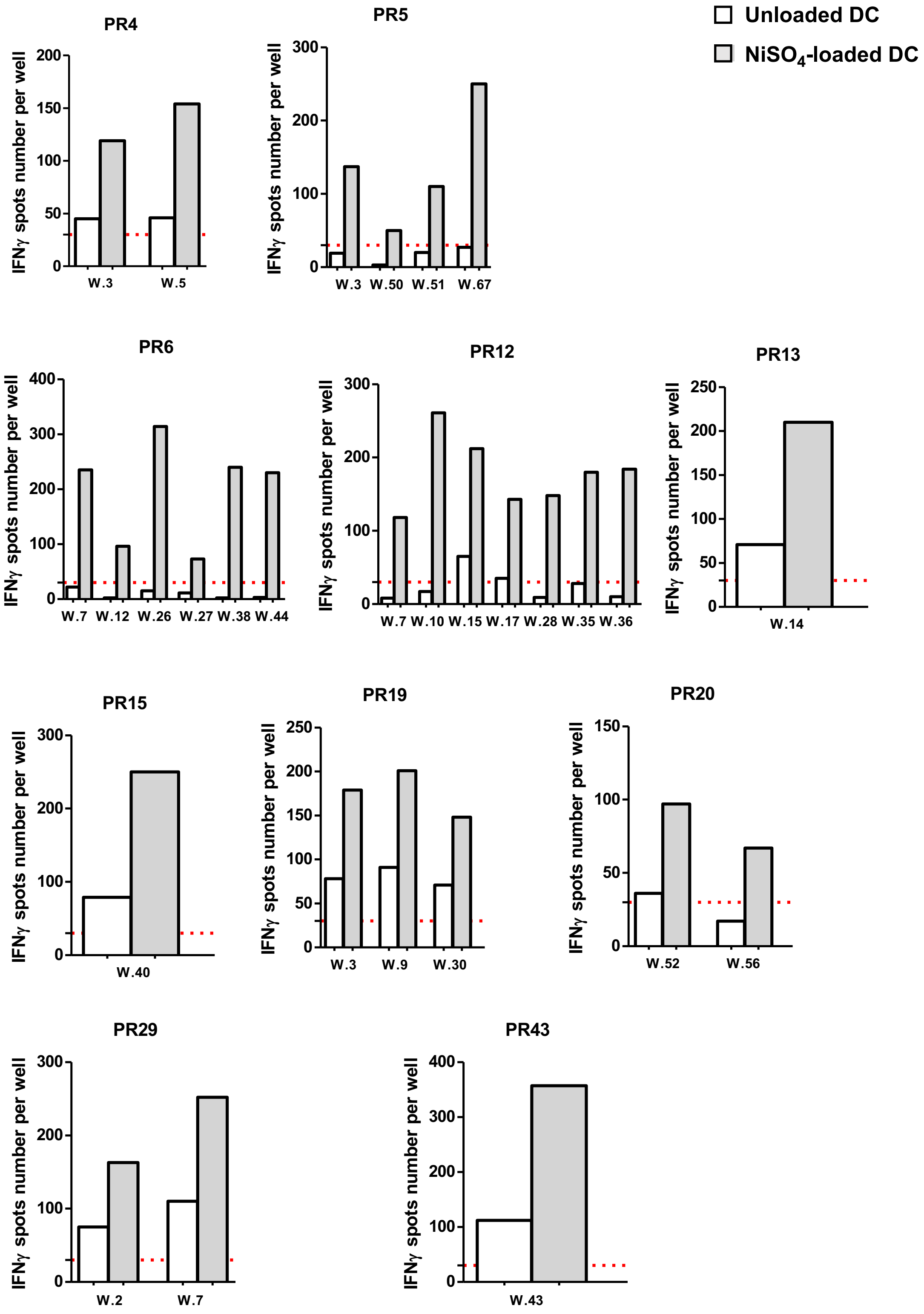

Supplementary data 3

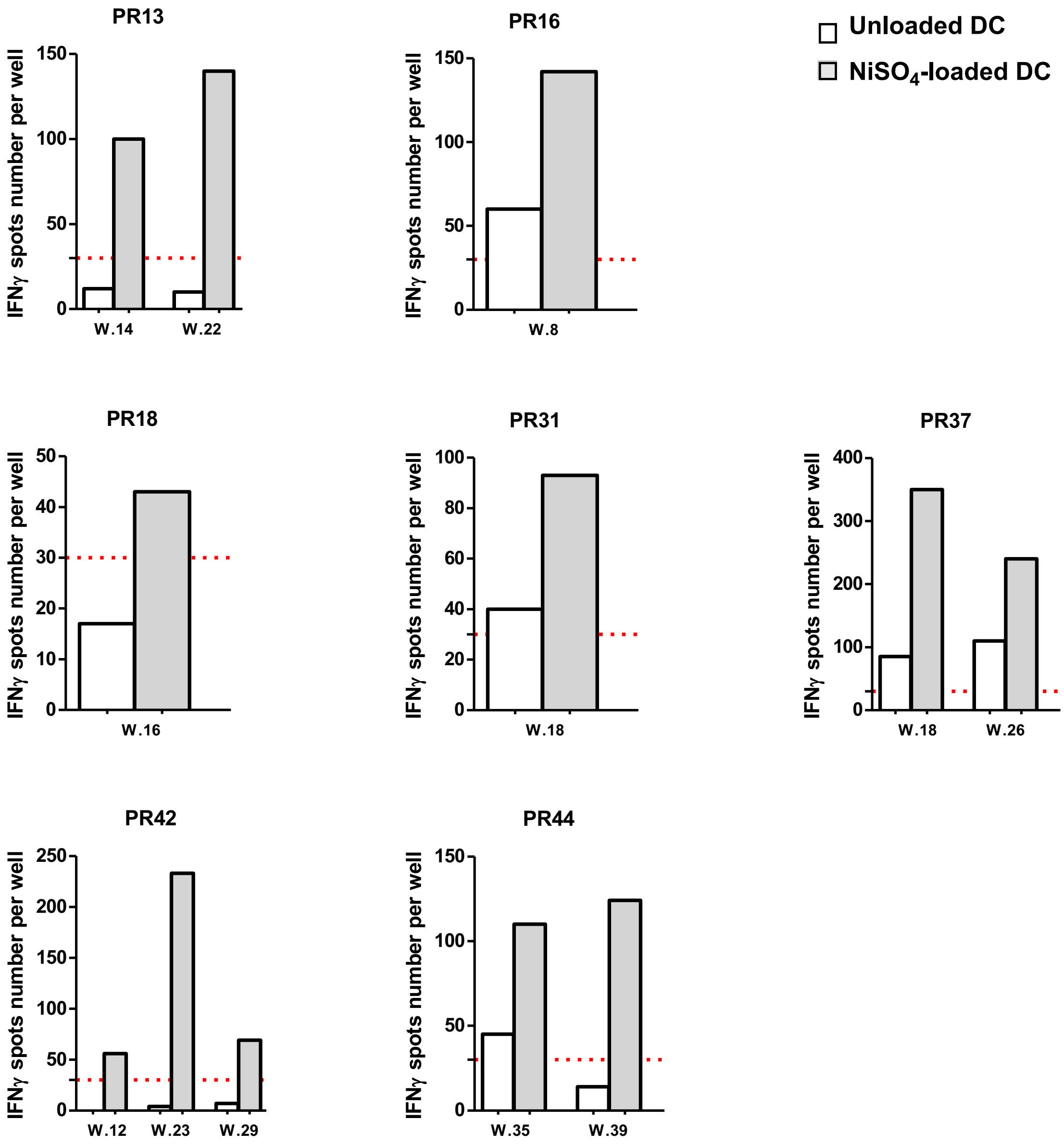

Supplementary data 4

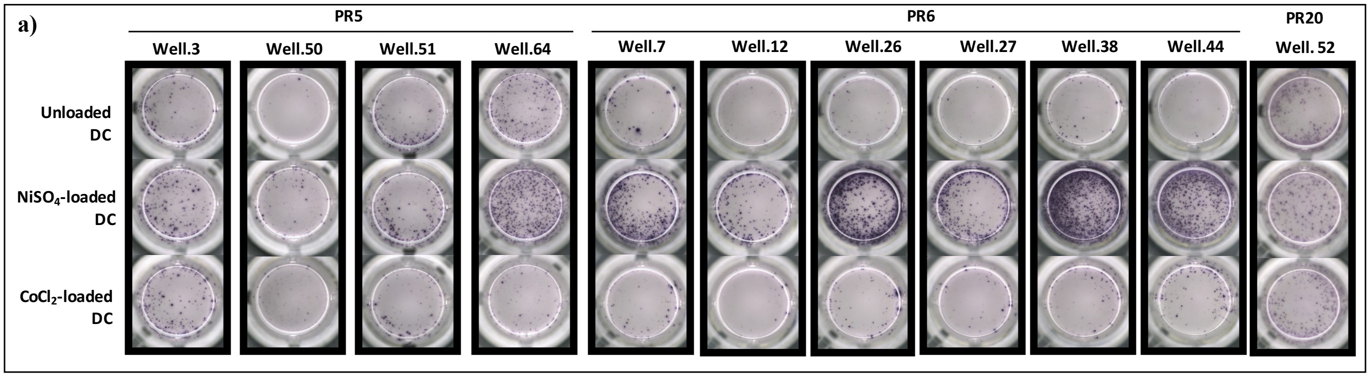

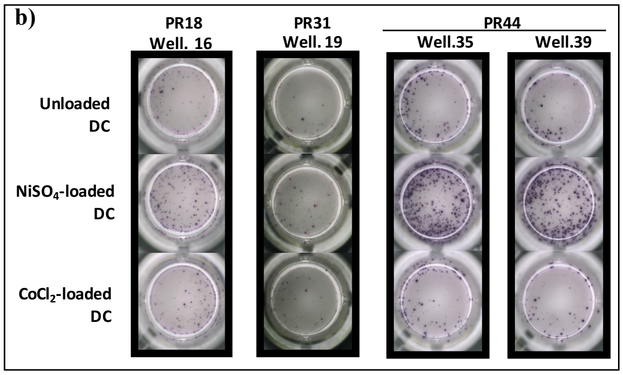

Supplementary data 5

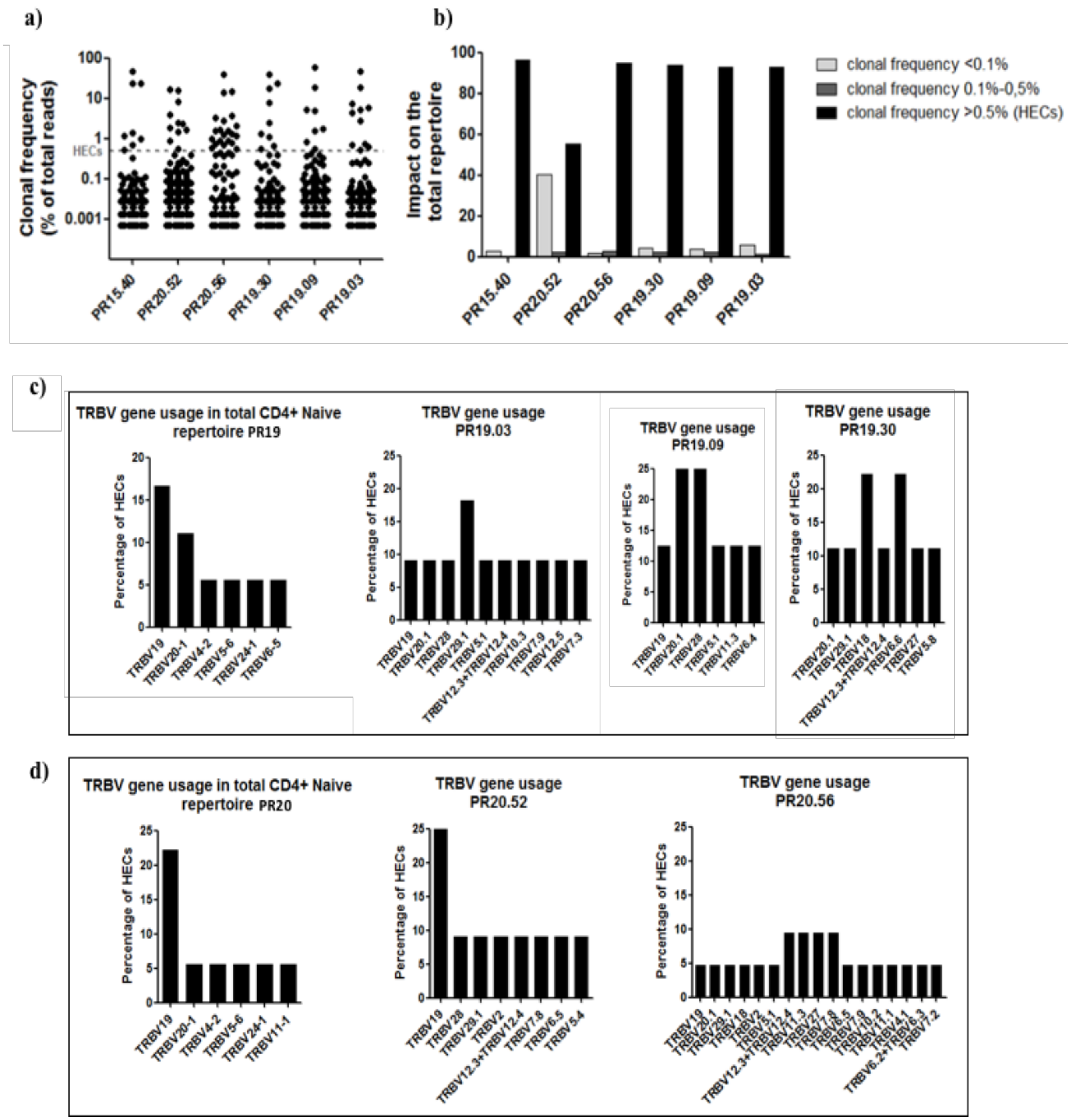

Supplementary data 6

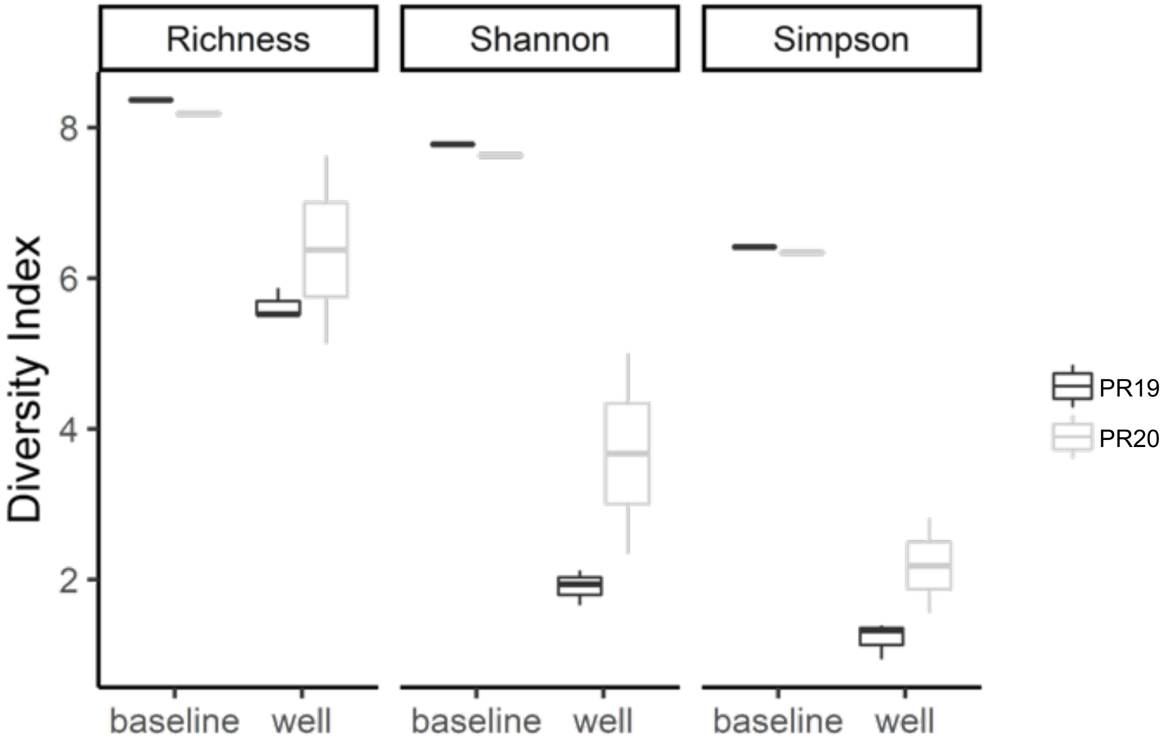

Supplementary data 7

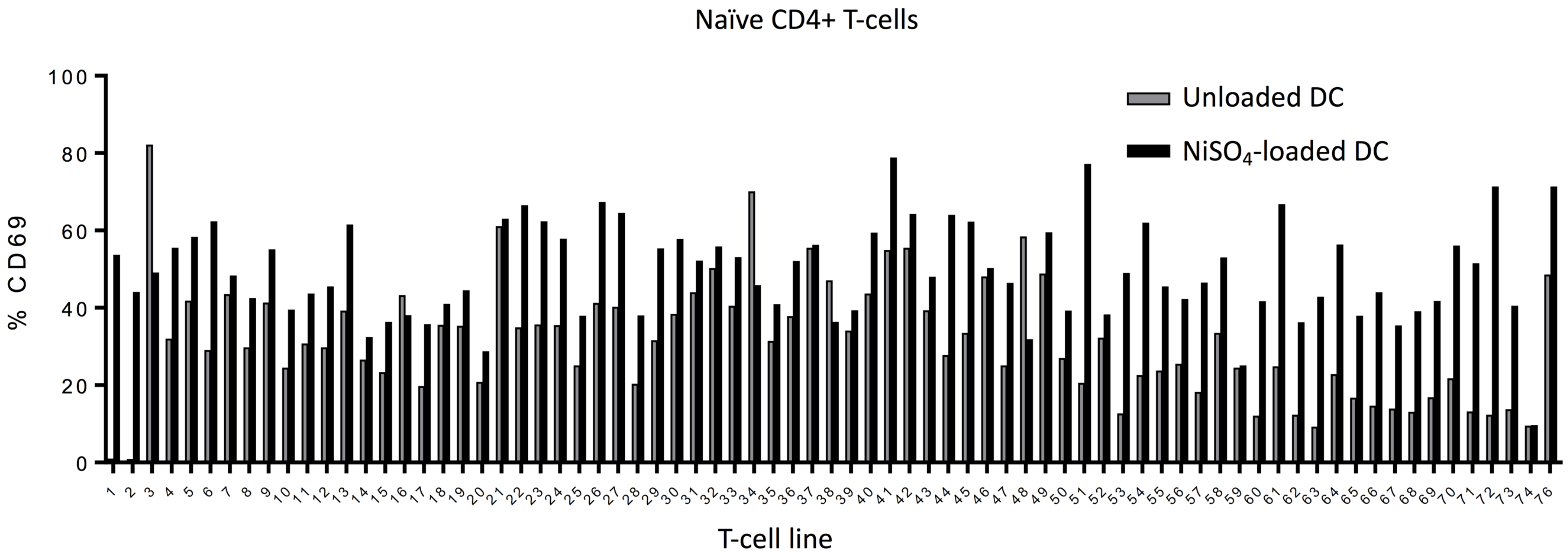

Supplement: Supplementary Data 1 — Gating strategy and cell viability for MoDC/T-cells co-culture. (a) IP (propidium iodide) staining of MoDC and CD8 + T cells after 7 days of coculture. (b) IP staining of CD8 + T cells detected after 3 round of stimulation. Purified naïve CD8+ T were seeded in multiple wells and stimulated weekly by autologous DCs previously loaded with nickel. After three rounds of stimulation, the specificity of the T-cell lines was tested by IFN-γ Elispot assays. One positive T-cell line was randomly chosen and its viability was assessed with IP staining. [file Data_Sheet_1.PDF]
